# Spontaneous resolution of severe, symptomatic mesocolic panniculitis: a case report

**DOI:** 10.1186/1471-230X-12-59

**Published:** 2012-06-06

**Authors:** Aurélie Daumas, Serge Agostini, Julia Villeret, Philippe Ah-Soune, Olivier Emungania, Brigitte Granel

**Affiliations:** 1 Service de médecine interne, Assistance Publique–Hôpitaux de Marseille (AP–HM), Université AIX-MARSEILLE, hôpital Nord, chemin des Bourrely, 13915, Marseille cedex 15, France; 2 Service d’imagerie médicale, hôpital privé Beauregard, 23 rue des Linots, 13012, Marseille, France; 3 Service d’anatomocytopathologie, AP–HM, Université AIX-MARSEILLE, hôpital Nord, chemin des Bourrely, 13915, Marseille cedex 15, France; 4 Service de gastroentérologie, AP–HM, Université AIX-MARSEILLE, hôpital Nord, chemin des Bourrely, 13915, Marseille cedex 15, France; 5 Service de chirurgie digestive, AP–HM, Université AIX-MARSEILLE, hôpital Nord, chemin des Bourrely, 13915, Marseille cedex 15, France; 6 Service de médecine interne, gériatrie et thérapeutique, hôpital de la Timone, 264 Rue Saint Pierre 13385, Marseille cedex 05, France

**Keywords:** Mesenteric panniculitis, Mesenteric lipodystrophy, Sclerosing mesenteritis, Mesocolic panniculitis

## Abstract

**Background:**

Mesenteric panniculitis is a rare chronic fibrosing inflammatory disease that typically affects the adipose tissue and mesentery of the small intestine but may also affect the mesosigmoid and the mesocolon. The pathology of this disease remains unclear despite association with some malignancies or inflammatory disorders. We report a case of mesocolic panniculitis and a literature review of its clinical presentation, imaging findings, associated conditions and treatment options.

**Case presentation:**

A 64 year-old Caucasian man was admitted to the gastroenterology department for severe weakness, left lower quadrant abdominal pain, weight loss and diarrhoea. Physical examination revealed a palpable firm mass occupying the entire left part of the abdomen. Abdominal CT-scan showed fatty infiltration of the mesosigmoid and left mesocolic fat which was strongly suggestive of panniculitis. Laparoscopic surgery revealed an inflamed and edematous mesocolon and mesosigmoid; the sigmoid mucosa appeared petechial which was suggestive of venous ischemia. Histological examination of surgical biopsies revealed mesocolic panniculitis. Despite exhaustive investigation, no associated conditions were found and the cause was classified as idiopathic. Surprisingly, the patient clinically improved without therapeutic intervention other than supportive care.

**Conclusion:**

Although mesenteric panniculitis is most often a radiographic diagnosis without clinical symptomatology, it can also present with significant general status alteration. We report a case of mesocolic panniculitis complicated by development of an inflammatory mass associated with ischemic colitis. Mesenteric panniculitis is a difficult diagnosis to make which typically requires histologic confirmation. The overall prognosis is good with supportive treatment.

## Background

Mesenteric panniculitis (MP) is a benign fibro-inflammatory process involving adipose tissue of the mesentery which is characterized by the presence of fat necrosis, chronic inflammation and fibrosis. This term is also improperly used to characterize the involvement of the mesosigmoid and the mesocolon, peripancreatic and omental fat and less frequently retroperitoneal or pelvic fat. We report here an unusual case of MP located in the mesentery of the sigmoid and left colon presenting with ischemic colitis. This rare observation of mesocolic panniculitis (MCP) was notable for the reversible ischemic colitis and abdominal pseudotumoral mass related to this condition. Here we highlight the differential diagnosis of this rare disease and review the published literature.

## Case presentation

**Figure 1:**
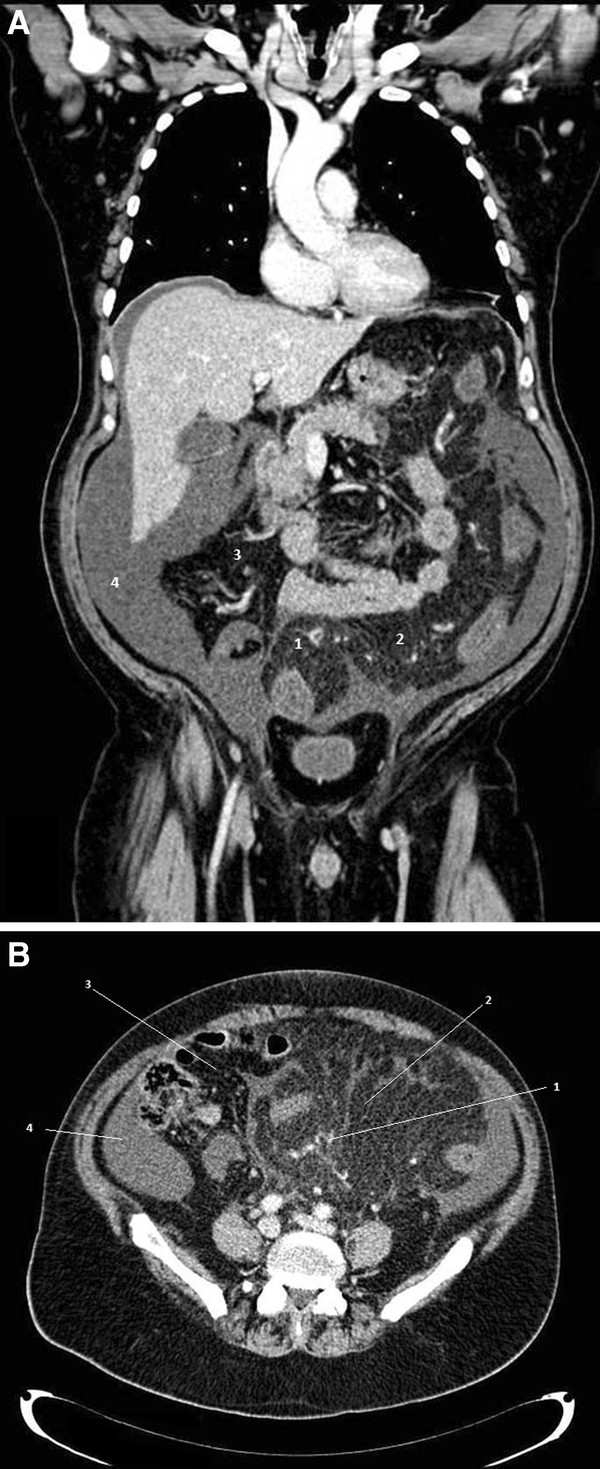
**A and B**: **CT-scan showing mesocolic panniculitis.** Contrast-enhanced coronal **(1A)** and axial **(1B)** CT scan showing mesocolic enlargement with hypervascularization (1), increased density of the mesocolic fat (2) compared with mesenteric fat (3) and ascites (4) suggestive of mesocolic panniculitis of the sigmoid and the ascending colon.

A 64 year-old Caucasian man was admitted to the gastroenterology department for severe general weakness, left lower quadrant abdominal pain, weight loss (more than 10 % of his normal weight) and diarrhoea for four weeks prior to admission. There was no history of melena, bloody diarrhea or fever. His past medical history included only type 2 diabetes for which he received oral antidiabetic agents. He had no cardiovascular risk factors and did not consume alcohol. He had travelled to Tunisia one year previously. Physical examination revealed a palpable firm mass occupying the entire left part of the abdomen without evidence of bowel obstruction. Blood tests showed an elevated C-reactive protein level (80 mg/L, normal range < 5 mg/L) without leucocytosis and a decreased potassium level. Hepatic and pancreatic enzyme levels and renal function tests were normal. The hepatitis B, C and HIV serological tests were negative. Blood and stool bacterial cultures as well as Clostridium difficile toxin A and B were all negative. Contrast-enhanced thoraco-abdominal CT-scan showed a thickening of the wall of the sigmoid and ascending colon without contrast enhancement, an increased density of the thickened mesosigmoid and mesocolon and ascites, all of which suggested the diagnosis of MCP (Figures 1A and 1B). The intra-abdominal vessels were patent on vascular contrast CT-scan findings. Complete ileo-colonoscopy revealed nonspecific inflammatory changes of the sigmoid and the ascending colon. Histological analysis of multiple colonic biopsy specimens showed changes compatible with ischemic colitis. Analysis of ascitic fluid revealed chylous ascites without malignant cells. Tests for Mycobacterium tuberculosis infection in cultures of sputum, ascites, blood and stool were negative. Tumor markers were all in the normal biological range and a search for an underlying auto-immune disease was negative. Trophyrema Whipplei PCR analysis performed on blood, saliva and stools was negative. Serum immunoglobulins (Ig) levels and including IgG4 subclass level were in the normal range.

**Figure 2:**
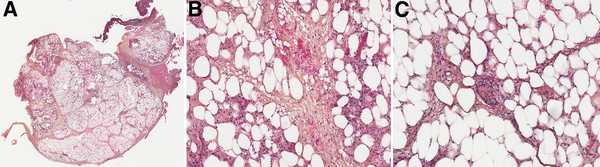
**A, B and C: Histological sections of mesocolic panniculitis.** Microscopic sections 2A (HES × 0.6) and 2 C (HES × 10) showing fibrous septa. Microscopic section 2B (HES × 10) showing small lymphocytes around capillaries and focal steato-necrosis with lipid-laden macrophages.

**Figure 3:**
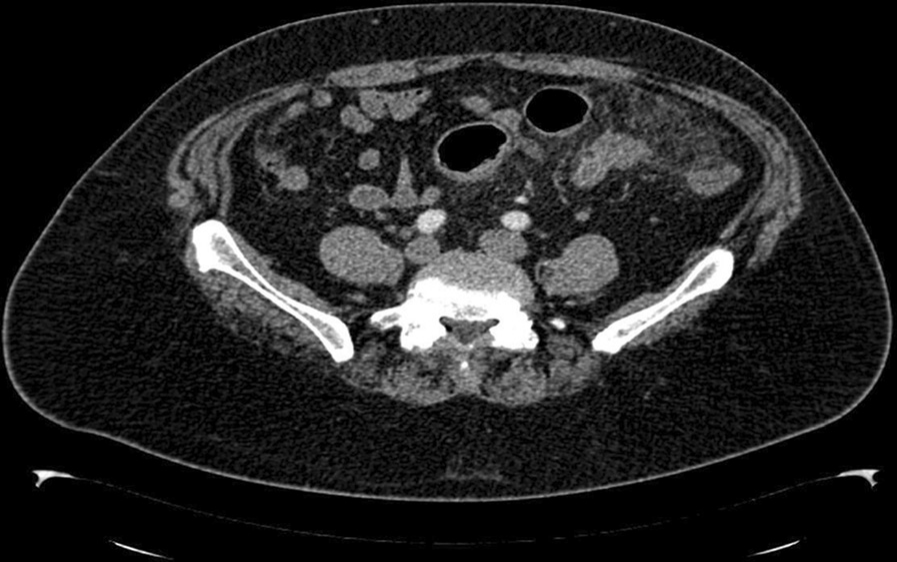
**CT-scan findings of mesocolic panniculitis one year later.** Axial contrast-enhanced abdominal CT scan performed one year later showed significant improvement in the radiologic appearance of the mass with regression in size and disappearance of inflammatory signs in comparison to the first study.

To definitely eliminate infectious or neoplastic disorders, a laparoscopic surgery was performed. The mesosigmoid and mesocolon were markedly thickened and retractile. The normal architecture was lost and was replaced with an irregular nodular mass which was difficult to move. The adjacent bowel wall was thickened, edematous and congested, with a petechial aspect. There was a moderate amount of serous ascites in the peritoneal cavity. No neoplasia, diverticula, abscesses or enlarged lymph nodes were identified. Histological examination of surgical mesocolon biopsies showed scattered areas of steatonecrosis with lipid-laden macrophages, lymphocytes and congested capillaries (Figure 2A, 2B and 2C). There were no granulomas, vasculitis or malignancy. All PCR and cultures for bacteria, mycobacteria, fungi and Whipple’s disease were negative. With only supportive care and without the administration of immunosuppressive therapies, the diarrhea resolved and the patient began to gain weight, with overall symptomatic improvement. After one year of follow-up, the patient remained in good health and the abdominal mass decreased in size. Abdominal CT-scan performed one year after the diagnosis confirmed the decrease in size of the MCP and the disappearance of ascites (Figure 3).

## Conclusions

MP is a benign, tumor-like mesenteric lesion, formed as result of chronic inflammation of adipose tissue; the etiology is unknown. In the literature, the disease is named with a number of synonyms: mesenteric lipodystrophy and sclerosing or retractile mesenteritis, each of which is a histologic variant based on the relative amounts of inflammation, fat necrosis and fibrosis [1,2]. While a large series of cases of small-bowel MP has been reported [1,3-14], this process rarely involves the mesentery of the large intestine, as was observed in this case.

MP has a slight male predominance and commonly occurs in late adulthood [1,3,4]. The symptoms of the disease are varied, and mostly include intermittent abdominal pain (34.6%), presence of abdominal mass (30.8%), nausea/vomiting, weight loss, bowel habit changes, with or without fever [1,2,11,12,15,16]. In the study by Akram et al. [4], common symptoms included abdominal pain in 70% of cases, diarrhoea in 25% and weight loss in 23%. Symptoms associated with MP can be caused by direct mechanical effects of the abdominal mass encasing the bowel, blood vessels and lymphatics, which can result in partial bowel obstruction [8], ischemia [6] or chylous ascites [17]. As recently reported by Van lingen et al. [14], MCP should be considered as a possible cause of unexplained colitis but can also manifest as ischemic colitis. The mechanism of ischemic colitis is thought to be secondary to venous insufficiency as opposed to arterial ischemia as evidenced by the absence of thrombosis of mesenteric arteries [13]. We hypothesize that severe edema of the mesocolon and mesosigmoid related to MCP induced venous ischemia which caused ischemia of the sigmoid mucosa. In the article by Akram et al. [4], 14% of MP patients were found to have chylous ascites, as was also the case in our observation. The reported clinical presentation of MP of the small intestinal mesentery or the large intestine is not significantly different. Generalized weakness with weight loss and severe bowel dysfunction are symptoms described in both [8,9]. Usually, the diagnosis of MP is made after several months’ history of recurrent abdominal pain, continuing weight loss, altered bowel habits and the presence of a palpable abdominal mass [11,15,16]. However, it is important to recall that most cases of MP (up to 92% in the study by Daskalogiannaki et al. [5]) are asymptomatic and are incidentally detected on abdominal CT performed for unrelated conditions.

Abdominal CT and magnetic resonance imaging (MRI) play important roles in suggesting the accurate diagnosis and can be used to distinguish MP from other diseases with similar imaging features such as carcinomatosis, carcinoid tumor, lymphoma or desmoid tumor. The “fat ring sign” (preservation of the densitometric values of fat near the mesenteric vessels) and the finding of “tumor pseudocapsule” (hyper-attenuated stripe partly surrounding the mass) are considered to be suggestive of mesenteric involvement [18]. However, in some cases, the differential diagnosis (Lymphoma / Carcinoid tumors / Desmoid tumors / Carcinomatosis / Peritoneal mesothelioma / Retroperitoneal sarcoma / Amyloidosis / IgG4-related retroperitoneal fibrosis / Infectious diseases (including tuberculosis, histoplasmosis and Whipple’s disease) / Reaction to adjacent or chronic abscess / Chronic inflammation due to foreign body) cannot be narrowed by imaging alone and biopsy with histological analysis is required.

A recent study by Zissin et al. [19] suggested that positron emission tomography (PET) with 18 FDG may be used to differentiate between benign and neoplastic processes of the mesentery. According to the authors, PET can be used to correctly exclude mesenteric tumor involvement when no FDG uptake was observed associated with typical CT features of MP. However, a recent case report [17] described a patient with symptoms and CT findings of MP and a negative uptake of FDG on PET, who was subsequently found to have a lymphoma on biopsy.

Thus, a biopsy should be performed in symptomatic patients to confirm the diagnosis of MP, because imaging specificity is limited since a broad differential diagnosis exists for mass lesions of the mesentery, particularly mesenteric tumour involvement [5].

The pathological mechanism of MP is not clearly known but would appear to involve a nonspecific response to a wide variety of stimuli. Daskalogiannaki et al. [5] reported the co-existence of MP and various neoplastic diseases not involving the mesentery in up to 69% of patients with MP, most commonly urogenital malignancies and digestive carcinoma or lymphoma but also extra-abdominal malignancies such as lung and breast carcinoma. Association of MP and malignancy was previously indicated in the literature [1,9,20-23] with 30% of patients with MP having an underlying malignancy such as lymphoma, for example in 8/53 patients (4.24%) [22]. The pathogenic link between MP and malignant disease remained unclear and a possible paraneoplastic response was suggested [5,21]. Besides neoplastic disorders, Emory et al. [1] reported a series of MP in which 4.76% of patients had a history of abdominal trauma or surgery, suggesting an inflammatory post-traumatic fat response [24]. Furthermore, the disease was also related to other factors such as infectious [25] and autoimmune diseases [5], vasculitis [20], vascular insufficiency, cirrhosis, peptic ulcer, pancreatitis or abdominal aortic aneurysm [5]. Finally, MP can occur without pre-existing or co-existing disease and can be classified as idiopathic, as was the case in our observation.

MP has been included in the field of fibrotic disorders such as retroperitoneal fibrosis, sclerosing cholangitis, Riedel’s thyroiditis and orbital pseudo tumour [11]. The pathogenesis of these fibrotic disorders may be related as they share common histological features and may have both elevated serum IgG4 levels and IgG4-producing plasma cell expansion in affected organs with fibrotic or sclerotic changes [26]. In our case, the patient did not have elevated serum levels of IgG4. IgG4-related disease is a systemic syndrome classically considered as a distinct entity belonging to the differential diagnosis of MP. However, they can sometimes mimic one other. Both diseases rarely co-exist as evidenced by two recent Japanese cases reporting IgG4-related sclerosing mesenteritis [27,28]. Moreover, a recent review on the subject proposes adding sclerosing mesenteritis to the list of diseases associated with IgG4 disorders [29]. Further studies will be required to confirm such an association.

Several therapies have been proposed to treat MP but no consensus has been established since no one therapy has been shown to be superior to the others. Treatment may include steroids [4,12,16,30,31], thalidomide, cyclophosphamide [32], progesterone [33], colchicine [12], azathioprine [16,30,31], tamoxifen [4,16,31], antibiotics, or radiotherapy.

The surgical approach should be limited to biopsy of the mass; resection or bypass procedures are proposed only in cases of bowel obstruction or perforation [12]. Although Adachi et al. [10] found that MP of the colon seems to be more progressive with surgical treatment required more often than MP of the small intestine, our patient did not require surgery and had spontaneous improvement.

MP is a rare entity that occurs independently or in association with other disorders. This process mainly affects the mesentery of the small intestine but involvement of the colon is not uncommon. Diagnosis is a challenge despite the help of imaging tools, and histological analysis is necessary in some symptomatic cases in order to rule out treatable alternate etiologies. Overall the prognosis is usually good with supportive treatment, even in symptomatic cases like the one reported here.

## Consent

Written informed consent was obtained from the patient for publication of this case report and any accompanying images. A copy of the written consent is available for review by the Editor-in-Chief of this journal.

## Abbreviations

MP: Mesenteric panniculitis; MPC: Mesocolic panniculitis.

## Competing interests

The authors declare that they have no competing interest.

## Authors’ contribution

AD for design and drafting of the manuscript. SA for his expertise on imaging of the patient and his help in drafting the manuscript. JV for the histological diagnosis, the histological figures and the revision of the manuscript. PA-S for acquisition, analysis, interpretation of data and tight surveillance of the patient. OE for the macroscopic description of the mass, the surgical treatment and the revision of the manuscript. BG took in charge the patient until the final diagnosis, has been involved in drafting the manuscript and its revision, and gave final approval of the version to be published. All authors read and approved the final manuscript form.

## Pre-publication history

The pre-publication history for this paper can be accessed here:

http://www.biomedcentral.com/1471-230X/12/59/prepub
